# A rare case of pectus carinatum

**DOI:** 10.11604/pamj.2024.49.57.45387

**Published:** 2024-10-29

**Authors:** Nitin Shankarrao Madavi, Swati Parteti

**Affiliations:** 1 Department of Human Anatomy, Mahatma Gandhi Ayurved College, Hospital and Research Centre Salod (H) Wardha, Datta Meghe Institute of Higher Education and Research, Deemed to be University, Wardha, Maharashtra, India,; 2 Department of Kriya Sharir (Human Physiology), Government College of Ayurved and Hospital Nanded, Maharashtra University of Health Sciences Nashik, Maharashtra, India

**Keywords:** Marfan syndrome, pectus excavatum, pectus cavernatum

## Image in medicine

The patient is an 11-month-old infant boy who has a rare congenital chest abnormality of the anterior chest wall known as *pectus carinatum. Pectus carinatum*, sometimes known as “Pigeon chest,” is a congenital distortion of the anterior chest wall that is similar to *pectus excavatum*. The disease is identified by an outward protrusion of the sternum or rib cage. The figure shows the right-sided chest wall anomaly, which was noticeable and caused mild discomfort and pain when lifting, sleeping, sitting, and standing. The abnormality was marked by a circle suggestive of *pectus carinatum*, which deformed over time as the patient matured. His parents were quite concerned that his chest abnormality would interfere with typical physical activity at home, and they were self-conscious about their infant boy's looks. The patient's parent was beginning to isolate the baby boy from friends and family and exhibiting signs of sadness. They stated that their primary concern for their baby boy was his family's emotional state of mind, which was closely tied to the cosmetic aspect of his chest wall malformation. The parents believed that surgery to treat the *pectus carinatum* was the next logical step. *Pectus carinatum* is a unique chest wall malformation that usually appears in childhood and gets more noticeable in puberty. *Pectus carinatum* is a structural abnormality of the chest wall that affects almost one in every thousand teens, and early detection allows for more noninvasive treatment choices.

**Figure 1:**
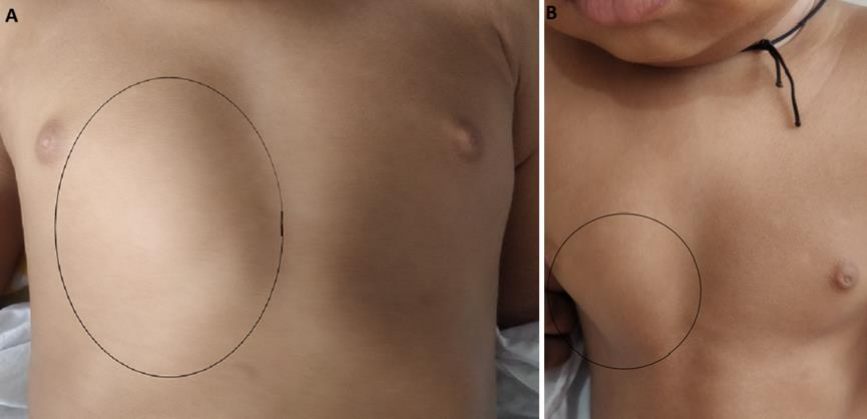
A) baby in lying position; B) baby in standing position (marked circle suggestive of *pectus carinatum*)

